# Seasonal data on Rose Bengal stained foraminifera in the head of Kongsfjorden, Svalbard

**DOI:** 10.1016/j.dib.2019.104040

**Published:** 2019-05-23

**Authors:** Olga Kniazeva, Sergei Korsun

**Affiliations:** a Saint-Petersburg State University, Russia; b Shirshov Institute of Oceanology RAS, Russia

**Keywords:** Benthic foraminifera, Arctic fjord, Seasonal dynamics

## Abstract

Current ‘Atlantification’ of the Arctic Ocean affects benthic communities leading to the changes in their structure and abundance. Such areas as Svalbard that are seasonally affected by Atlantic and Arctic water masses may give a possibility to preliminary estimate the response of benthic communities to short-term environmental changes and to evaluate their sensitivity. We have sampled Kongsfjorden for modern benthic foraminifera in three different seasons. The record includes data on the abundances of benthic foraminiferal species in the surface sediments (0–2cm). This data gives an insight into the seasonal dynamics of the near-glacial foraminiferal community of Kongsfjorden.

**Table:** Specifications table

Subject area	*Earth and Planetary Sciences*
More specific subject area	*Benthic Foraminifera*
Type of data	*Tables, figure*
How data was acquired	*Samples were collected during three expeditions to Kongsfjorden from 2015 to 2016 (in January, September and June), using box corer, stained with Rose Bengal; live and benthic foraminifera were identified to the species level and counted.*
Data format	*Tables with densities of live foraminifera; a table with station list, coordinates, water depth.*
Experimental factors	*Sampling was carried out in January 2015, September 2015 and June 2016 to cover different seasons and get a grasp on seasonal changes in the foraminiferal community*
Experimental features	*The surface sediment was collected with a box corer. Samples were stained with Bengal Rose and washed on a 63um sieve.*
Data source location	*Kongsfjorden, Svalbard archipelago (GPS coordinates are provided in the table)*
Data accessibility	*The data are available with this article*
Related research article	*Jernas, P., Klitgaard-Kristensen, D., Husum, K., Koç, N., Tverberg, V., Loubere, P., … & Gluchowska, M. (2018). Annual changes in Arctic fjord environment and modern benthic foraminiferal fauna: evidence from Kongsfjorden, Svalbard. Global and planetary change, 163, 119–140.*

**Table:** 

**Value of the data** • The data traces the seasonal dynamics of living benthic foraminiferal assemblages based on the Rose Bengal staining. • The data keeps record of foraminiferal response to the changing throughout the year water masses. • The data provides an insight into the structure of near-glacial foraminiferal community.

## 1 Data

Glaciated subpolar fjords are widely represented in the Northern Hemisphere. These are dynamic systems that are characterized by specific circulation [1] and are influenced by glacial meltwater runoff that brings vast amount of rapidly accumulating mineral matter [2], [3]. Both these factors influence fjord benthic communities.

**Fig. 1:**
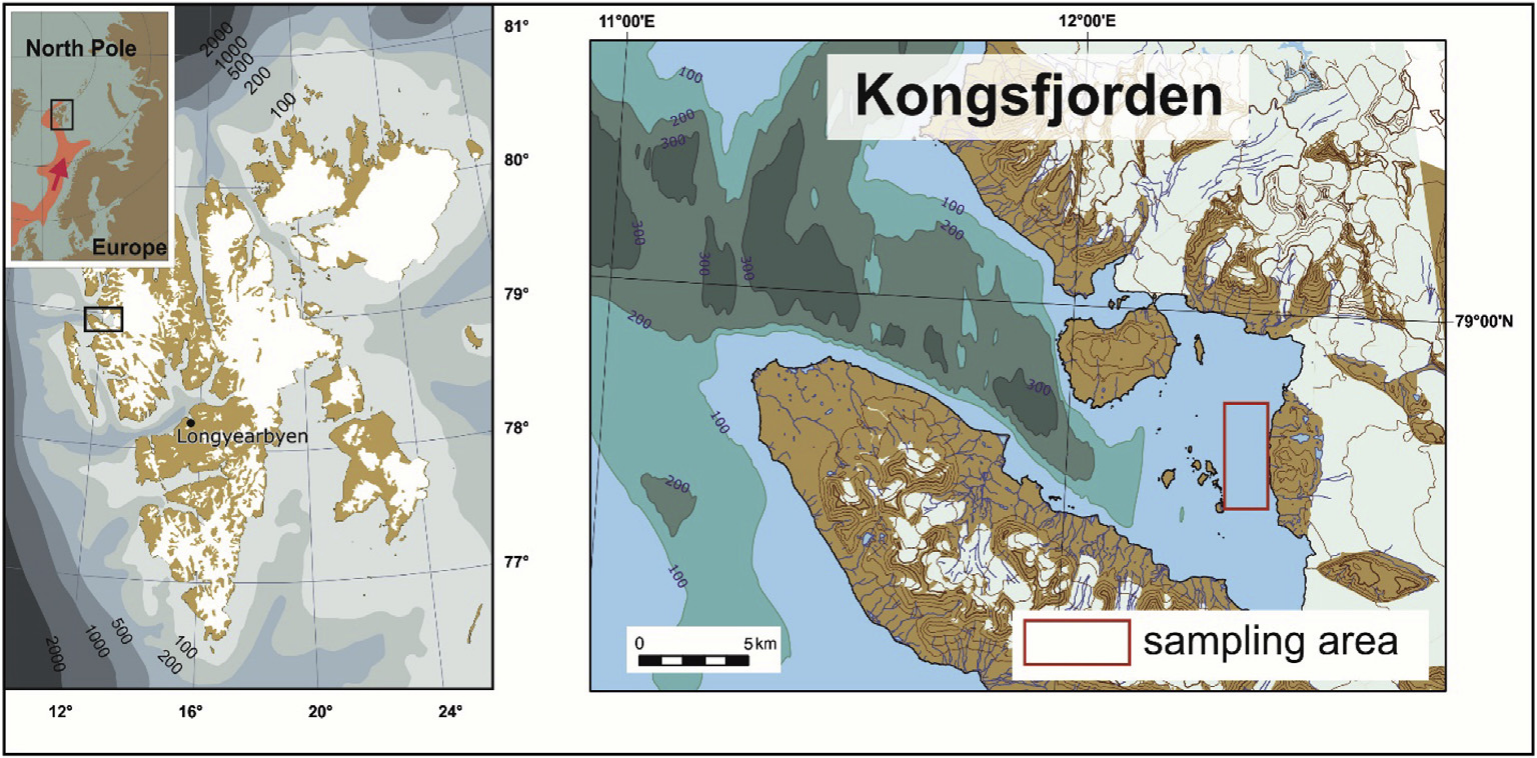
Location of the sampling area. Left - Svalbard. Right - Kongsfjorden.

Kongsfjorden is a glacially fed fjord located in the western part of West Spitsbergen (Fig. 1). The fjord is influenced by both Atlantic and Arctic water masses that mix and interchange throughout the year [4], [5]. Benthic foraminiferal community of Kongsfjorden has been investigated by a number of authors. However, in most of the previous studies sampling was carried out in late spring – early autumn and did not include winter months [6], [7]. Still little is known of the changes it undergoes interannually.

Here we present a two-year dataset that covers three subsequent seasons: winter 2015, autumn 2015 and spring 2016 (Table 1). The data are living foraminiferal abundances normalized to 10cm^3^ of sea-floor surface sediment (Table 2, Table 3, Table 4).

**Table 1:** Station list.

January 2015				September 2015				June 2016			
St.	Latitude N	Longitude E	Water depth [m]	St.	Latitude N	Longitude E	Water depth [m]	St.	Latitude N	Longitude E	Water depth [m]
7	78° 55.600′	12° 24.500′	51.8	1	78° 55.480′	12° 23.030′	44	1	78° 55.490′	12° 24.000′	47.7
8	78° 55.600′	12° 23.600′	51.5	2	78° 55.520′	12° 23.790′	49.4	2	78° 55.500′	12° 21.190′	42.6
9	78° 55.640′	12° 21.600′	54	3	78° 56.490′	12° 24.110′	53.6	3	78° 56.500′	12° 21.000′	54.1
10	78° 55.700′	12° 22.400′	51	4	78° 56.500′	12° 21.160′	62.2	4	78° 57.530′	12° 21.000′	62
11	78° 57.620′	12° 24.300′	61.5	6	78° 57.530′	12° 24.140′	62.2	5	78° 56.440′	12° 23.940′	48.4
12	78° 57.470′	12° 21.080′	59	7	78° 56.520′	12° 21.100′	57	6	78° 57.560′	12° 23.980′	64.8
				8	78° 57.470′	12° 20.070′	57

**Table 2:** Density of living benthic foraminifera from sediment-surface samples retrieved in January 2015.

Original station_no	7A	7B	8A	8B	8C	9A	9B	9C	10A	10B	10C	11A	11B	11C	12A	12B	12C
Area	1					2						6			4		
*Adercotryma glomeratum*				1.4			5.3			3.2						2.6	
*Ammodiscus* sp.																	
*Ammotium cassis*																	
*Astrononion hamadaense*																	
*Bolivina* sp.				1.4											3.6		
*Buccella frigida*																	
*Cassidulina reniforme*	168.8	153.6	182.5	115.0	155.7	42.6	273.2	78.3	147.3	95.8	4.4	95.6	14.4	3.2	89.3	68.3	58.1
*Cornuspira* sp.											1.4	1.6		1.0	1.3		
*Cuneata arctica*																	
*Dentalina* sp.								1.6	2.9								
*Elphidium bartletti*													2.9				1.1
*Elphidium excavatum subsp. clavatum*	153.6	94.1	127.2	62.3	149.1	226.8	572.7	185.3	135.5	546.3	296.9	74.5	63.4	42.8	55.5	15.8	51.4
*Epistominella* sp.				1.4								1.6		1.8	4.8		2.2
*Globobulimina* sp.																	
*Islandiella helenae*		2.2	2.2	5.5		3.9	15.8	1.6		41.5	7.3	69.7	6.5	27.3	59.1	63.5	32.3
*Labrospira crassimargo*				1.4		1.9	1.6	8.0		44.7	8.6	97.2	181.6	45.5	49.5	6.4	16.7
*Lagena* spp.	2.2	1.8		1.4					2.9		1.4				1.3		
*Lobatula lobatula*																	
*Miliolinella* spp.		4.3	2.2	5.5			21.2			3.2				1.0			
*Nonionellina labradorica*											1.4						
*Parafissurina* sp.												4.9	2.9	1.0	16.9	36.8	14.5
*Pullenia subcarinata*											1.4			2.7		2.6	
*Pyrgo williamsoni*	2.2	1.8	4.4	2.8		1.9	36.8	17.6	2.9	3.2		24.4		11.8	3.6	2.6	16.7
*Quinqueloculina* sp.			4.4			13.6	36.8	16.0			2.9		2.9	1.0	1.3	7.9	
*Quinqueloculina stalkeri*	82.2	73.6	26.3	18.8	15.4	33.0	152.4	76.7	173.0	6.4	5.8	27.5	31.7	21.8		15.8	
*Recurvoides turbinatus*																	
*Reophax arctica*			2.2									1.6				2.6	
*Reophax fusiformis*				1.4													2.2
*Robertina arctica*		3.2			6.6				2.9						1.3		2.2
*Silicosigmoilina groenlandica*	2.2			2.8			5.3				1.4		2.9	2.7	1.3	5.3	1.1
*Spiroplectammina biformis*	28.1	34.6	48.3	15.2	98.7	5.8	5.3	14.4	63.4	6.4	1.4	9.7	8.6	1.9			4.5
*Stainforthia* spp.	4.3	3.2	15.4	11.8	2.0	15.6	47.3	6.4	2.9	3.2	13.0	14.6	14.4	2.7	8.4	18.4	11.2
individual/10cm3	443.6	372.6	415.0	248.2	427.5	345.2	1173.4	405.7	533.6	753.9	347.5	423.0	332.3	168.3	297.1	248.6	214.4
no. of specimen	205	344	189	179	199	178	225	254	185	236	266	261	134	224	246	149	192
no. of taxa	8	11	10	16	6	9	12	10	9	10	13	12	11	15	14	13	13
% calcareous	94	91	88	92	77	98	99	94	88	93	97	74	43	72	83	95	89

**Table 3:** Density of living benthic foraminifera from sediment-surface samples retrieved in September 2015.

Station_no	1A	1B	1C	2A	2B	2C	4A	4B	4C	7A	7B	7C	8A	8C	3A	3B	3C	6A	6B	6C
*Adercotryma glomeratum*																				
*Ammodiscus* sp.																		0.7		
*Ammotium cassis*													0.9							
*Astrononion hamadaense*																				
*Bolivina* sp.	2.5											2.0						0.7		0.5
*Buccella frigida*																				
*Cassidulina reniforme*	281.7	164.3	23.8	4.9	51.4	59.2	94.0	86.6	66.0	224.9	369.8	150.0	51.8	57.9	62.9	49.4	77.7	28.2	4.5	16.6
*Cornuspira* sp.																				
*Cuneata arctica*										2.0		2.0			0.9		2.8			
*Dentalina* sp.			2.0					1.6		2.0	4.9			1.2			0.9			0.5
*Elphidium bartletti*				0.5			1.7					2.0			0.9			2.2		0.5
*Elphidium excavatum subsp. clavatum*	17.1	115.4	138.8	18.3	7.7	98.6	172.3	81.4	8.8	173.6	261.3	165.7	18.5	34.5	12.2	41.9	43.5	46.6	7.4	37.0
*Epistominella* sp.							1.7			3.9								0.7		0.9
*Globobulimina* sp.																				
*Islandiella helenae*	14.8	3.0	3.9				1.4	4.2	4.4	31.6	78.9	29.6	14.8	13.6	0.9		0.9	3.7	0.5	5.5
*Labrospira crassimargo*	4.9		2.0		1.6		3.5	4.2	12.1	53.3	83.8	41.4	17.6	9.9				3.7	0.6	3.7
*Lagena* spp.																				
*Lobatula lobatula*																		0.7	0.5	
*Miliolinella* spp.							1.7													
*Nonionellina labradorica*		1.5		0.5						7.9		2.0	4.6	1.2	7.4	0.7		0.7	0.6	1.4
*Parafissurina* sp.										2.0		2.0						1.5	0.2	0.9
*Pullenia subcarinata*																		0.7	0.2	
*Pyrgo williamsoni*	14.8	7.4	15.8	0.5	1.6	2.0			1.3	2.0	4.9	9.9	1.8	1.2	1.8	2.2			0.2	
*Quinqueloculina* sp.	2.5						1.7	1.6					2.8	1.2	2.8		3.7			0.9
*Quinqueloculina stalkeri*	17.3	11.8	5.9	1.4	4.7	13.9	5.2	1.6	6.7	2.0	4.9	2.0	5.5	3.7	15.7	1.5	22.2	1.5	0.4	0.5
*Recurvoides turbinatus*																				
*Reophax arctica*													0.9							
*Reophax fusiformis*																				0.5
*Robertina arctica*												2.0	0.9	1.2					0.2	0.5
*Silicosigmoilina groenlandica*	12.3	5.9	5.9					1.6	1.3			2.0						0.7		0.5
*Spiroplectammina biformis*	219.4	164.3	137.0	4.4	179.8	161.7	22.6	24.3	37.7	41.4	29.6	15.8	12.9	34.5	45.3	21.7	5.9	17.8	1.8	5.5
*Stainforthia* spp.	4.9	4.4	5.9	5.3	17.1	11.8	1.7	3.2	2.7	7.9	14.8	5.9	12.9	8.6	12.2	2.2		5.9	0.5	5.5
individual/10cm3	592.2	477.9	340.9	35.8	263.8	347.2	307.8	210.2	141.1	554.3	853.1	434.0	146.1	168.9	163.1	119.7	157.5	116.2	17.3	81.4
no. of specimen	302	323	277	149	209	176	182	206	158	281	173	220	158	137	293	172	219	157	112	176
no. of taxa	11	9	10	8	7	6	11	10	9	13	9	15	13	12	11	7	8	16	13	17
% calcareous	62	66	59	88	31	53	92	86	65	83	87	86	78	74	72	82	95	81	86	88

**Table 4:** Density of living benthic foraminifera from sediment-surface samples retrieved in June 2016.

Station_no	1A	1B	1C	2A	2B	3A	3B	3C	4A	4B	4C	5A	5B	5C	6A	6B	6C
*Adercotryma glomeratum*															1.7		
*Ammodiscus* sp.																	
*Ammotium cassis*																	
*Astrononion hamadaense*																0.8	
*Bolivina* sp.						1.3			0.5						0.9		
*Buccella frigida*									0.5	1.4						0.8	1.0
*Cassidulina reniforme*	181.5	227.1	347.3	225.7	115.6	24.6	84.1	48.4	2.2	13.2	14.3	149.4	78.1	181.5	9.5	14.8	16.2
*Cornuspira* sp.									1.4		0.4				3.5	0.8	
*Cuneata arctica*																	
*Dentalina* sp.															0.9		
*Elphidium bartletti*							3.3			0.5				1.6	6.9	1.6	1.0
*Elphidium excavatum subsp. clavatum*	137.3	416.4	171.3	158.6	99.0	127.0	29.2	199.5	29.2	15.8	32.5	187.8	121.4	296.8	16.4	26.4	21.4
*Epistominella* sp.											0.4					0.8	
*Globobulimina* sp.														1.6			
*Islandiella helenae*	3.9		2.6	4.3	2.2	3.9	9.9	9.7	1.9	15.3	7.2			4.9	13.0	19.8	17.2
*Labrospira crassimargo*			2.6	16.1		5.2	3.3	3.2	7.8	8.4	7.2		2.8		1.4	9.7	2.5
*Lagena* spp.									0.5								
*Lobatula lobatula*															1.7	0.8	
*Miliolinella* spp.		3.2		2.7								8.5		1.6			
*Nonionellina labradorica*				26.9	14.8	7.8	13.2	9.7	3.7	14.0	17.7	4.3		6.6	1.7	0.8	5.7
*Parafissurina* sp.								1.5		0.5	0.8				0.9	0.8	
*Pullenia subcarinata*																	
*Pyrgo williamsoni*	5.2			24.2	13.4			1.5	1.4	1.4		4.3		6.6	0.9		3.8
*Quinqueloculina* sp.							1.6		0.5		1.3		1.4	1.6			
*Quinqueloculina stalkeri*	1.3		5.2	5.4			3.3	4.5	0.5	0.9		8.5	1.4	4.9	0.9		
*Recurvoides turbinatus*				2.7													
*Reophax arctica*																	
*Reophax fusiformis*									0.5			4.3				1.6	
*Robertina arctica*				2.7	2.7				1.4	0.5	0.8						1.0
*Silicosigmoilina groenlandica*				5.4	4.3	1.3	1.6	6.5		0.5							
*Spiroplectammina biformis*	15.5	5.5	13.7	48.4	36.3	3.9	9.9	18.1	1.6	2.8	2.5	68.3	29.3	36.3	6.9	1.6	2.0
*Stainforthia* spp.	47.9	60.0	62.2	40.3	44.3	9.1	19.8	14.0	19.8	10.7	14.4	77.1	60.0	49.5	13.8	39.9	31.5
individual/10cm3	392.7	712.2	604.8	563.3	332.5	184.0	179.4	316.8	73.3	85.8	99.6	512.5	294.5	593.7	80.9	121.4	103.3
no. of specimen	303	240	268	223	260	142	267	208	242	184	236	120	211	360	104	146	126
no. of taxa	8	6	8	13	10	10	12	12	16	14	13	10	8	11	17	14	11
% calcareous	96	99	97	88	89	95	93	93	87	87	90	86	89	94	88	89	96

## 2 Experimental design, materials and methods

We sampled sea bottom sediments in the head of Kongsfjorden during three cruises with RV *Helmer Hanssen* (Fig. 1). Sampling with a 50 × 50cm box corer was carried out in January and September 2015 and June 2016 (Table 1). Three replicates of 0–2cm surface sediment of arbitrary volume (approx. 80–120ml) were taken from each box corer with the exception of station 7 where only two replicates were taken due to the partly disturbed sediment. Sediment was collected into cylindrical jars and the volume of each sample was calculated as a volume of cylinder (*V* = π*r^2^*h, where r - is the radius of the jar, h – is the height of the sediment in a jar). To distinguish living foraminifera we preserved samples with 96% alcohol solution of Rose Bengal dye. The staining period was 14 days minimum to provide the time for thorough staining of all living foraminifera. In the laboratory we washed the sediment on a sieve with 63um mesh size, kept it in 30% alcohol overnight to remove excess dye and dried it at 100 °C. We split samples to obtain practical aliquots containing 100 to 300 stained specimens. To split samples, we did not use a dry micro-splitter. Instead, we applied a splitting procedure which provides, in our experience, more reproducible results. We placed the dry residue on a glass plate and divided the heap with two cross cuts by a razor blade into four parts. The mixing of the opposite quarters gave us two identical halves. When necessary, the procedure was repeated to obtain 1/4, 1/8, or 1/16. Then an aliquot was processed as a whole. All 100 to 300 stained specimens were identified to the lowest possible taxonomical level and counted. The number of specimens in the sample was calculated as N*2^s^ (where N was the number of stained specimens counted in the split and S was the number of splits). Foraminiferal abundance was normalized to 10cm^3^ of wet sediment using the measured volume of the sediment in the sample bottle. Taxonomical guides were Höglund [8], Loeblich and Tappan [9], and Knudsen [10].

The data set comprises information organized in three sets of data:

1. Station list is including coordinates, months sampling, water depths (Table 1).

2. Surface sampling in the head of Kongsfjorden. This database comprises densities of live benthic foraminiferal species (size fraction >0.63 mm) sampled in January 2015, September 2015 and June 2016 (Table 2, Table 3, Table 4).

## Appendix Benthic foraminiferal species and genera considered

*Adercotryma glomeratum* (Brady, 1878).

*Ammodiscus* sp. Reuss, 1862.

*Ammotium cassis* (Parker, 1870).

*Astrononion hamadaense* Asano, 1950.

*Bolivina* sp. d'Orbigny, 1839.

*Buccella frigida* (Cushman, 1922).

*Cassidulina reniforme* Nørvang, 1945.

*Cornuspira* sp. Schultze, 1854.

*Cuneata arctica* (Brady, 1881).

*Dentalina* sp. Risso, 1826.

*Elphidium bartletti* Cushman, 1933.

*Elphidium excavatum subsp. clavatum* Cushman, 1930.

*Epistominella* sp. Husezima & Maruhasi, 1944.

*Globobulimina* sp. Cushman, 1927.

*Islandiella helenae* Feyling-Hanssen & Buzas, 1976.

*Labrospira crassimargo* (Norman, 1892).

*Lagena* spp. Walker & Jacob, 1798.

*Lobatula lobatula (Walker & Jacob, 1798)* = *Cibicidoides lobatulus*.

*Miliolinella* spp. Wiesner, 1931.

*Nonionellina labradorica* (Dawson, 1860).

*Parafissurina* sp. Parr, 1947.

*Pullenia subcarinata* (d'Orbigny, 1839).

*Pyrgo williamsoni* (Silvestri, 1923).

*Quinqueloculina* sp. Schwager, 1883.

*Quinqueloculina stalkeri* Loeblich & Tappan, 1953.

*Recurvoides turbinatus* (Brady, 1881).

*Reophax arctica* (Brady, 1881) = *Cuneata arctica*.

*Reophax fusiformis* (Williamson, 1858).

*Robertina arctica* d'Orbigny, 1846.

*Silicosigmoilina groenlandica* (Cushman) emend Loeblich & Tappan, 1953.

*Spiroplectammina biformis* (Parker & Jones, 1865).

*Stainforthia* spp. Hofker, 1956.
